# Contributions of the *C*-Terminal Helix to the Structural Stability of a Hyperthermophilic Fe-Superoxide Dismutase (TcSOD)

**DOI:** 10.3390/ijms10125498

**Published:** 2009-12-23

**Authors:** Sha Wang, Yong-Bin Yan, Zhi-Yang Dong

**Affiliations:** 1 State Key Laboratory of Microbial Resources, Institute of Microbiology, Chinese Academy of Sciences, Beijing 100101, China; E-Mail: wangs@mail.im.ac.cn (S.W.); 2 State Key Laboratory of Biomembrane and Membrane Biotechnology, School of Life Sciences, Tsinghua University, Beijing 100084, China

**Keywords:** Fe-superoxide dismutase, hyperthermophilic enzyme, thermostability, ion-pairing network, protein unfolding

## Abstract

Hyperthermophilic superoxide dismutases (SODs) are of particular interest due to their potential industrial importance and scientific merit in studying the molecular mechanisms of protein folding and stability. Compared to the mesophilic SODs, the hyperthermostable Fe-SODs (TcSOD and *Ap*SOD) have an extended *C*-terminal helix, which forms an additional ion-pairing network. In this research, the role of the extended *C*-terminus in the structural stability of TcSOD was studied by investigating the properties of two deletion mutants. The results indicated that the ion-pairing network at the *C*-terminus had limited contributions to the stability of TcSOD against heat- and GdnHCl-induced inactivation. The intactness of the *C-*terminal helix had dissimilar impact on the two stages of TcSOD unfolding induced by guanidinium chloride. The mutations slightly decreased the Gibbs free energy of the dissociation of the tetrameric enzymes, while greatly affected the stability of the molten globule-like intermediate. These results suggested that the additional ion-pairing network mainly enhanced the structural stability of TcSOD by stabilizing the monomers.

## 1. Introduction

Thermophilic enzymes, which are usually obtained from thermophiles, have extraordinarily high thermostability and exhibit their optimal catalysis at temperatures above 50 °C [1]. The ones with an optimal temperature above 80 °C are hyperthermophilic enzymes, which are of particular interest due to their potential industrial and commercial importance. Thermophilic enzymes also provide a valuable model system for studying enzyme evolution and mechanisms of protein stability, especially thermostability. Compared to its mesophilic homologues, many structural stabilizing factors have been characterized to contribute to the high thermostability of thermophilic enzymes, such as additional disulfide bridges, hydrogen bonds, ion pairs and hydrophobic interactions [1–4]. Among these factors, it is now well-known that thermophilic proteins show a statistically increased number of electrostatic interactions such as salt bridges [5]. Moreover, many thermophilic proteins are known to have higher oligomeric state when compared to their mesophilic homologues, which implies that the formation of high-order oligomers may be closely associated with their hyperthermostability [1,6–10]. It is also believed that the formation of the proper oligomeric structure is crucial to the catalysis and thermostability of the hyperthermophilic enzymes at high temperatures. However, the complexity in the folding and stability make it difficult to correlate the well-defined mechanisms in small proteins with the hyperthermostability of large oligomeric proteins.

Superoxide dismutases (SODs) are metalloenzymes that catalyze the disproportionation of superoxide radicals to protect cells from damaging by superoxide radicals [11]. SODs can be classified into four types depending on their metal selectivity, Cu/Zn-, Mn-, Fe- and Ni-SOD [12,13]. During the past 10 years, many Fe-SODs from hyperthermophiles have been discovered [7,14–17]. Among them, TcSOD, which was discovered from a hotspring in Tengchong, Yunnan (China) by construction of metagenomic library and DNA sequencing as described previously [17], has a high level of sequence homology (~80%) and structural similarity to the well-characterized thermostable Fe-SOD from *A. Pyrophilus* (*Ap*SOD) [14,17,18]. The inter- and intra-subunit interactions through ion pairs were found to be important to the stability of TcSOD tetramer and monomer [10,17].

**Figure 1.**
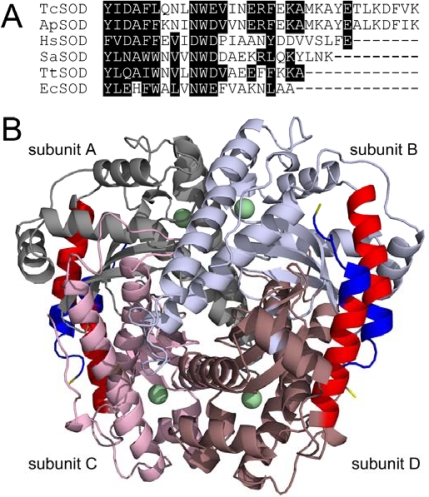
(A) Sequence alignment of the *C-*terminus of hyperthermophilic (TcSOD and *Ap*SOD) and thermophilic/mesophilic (HsSOD, SaSOD and TtSOD) SODs. *Ap*SOD, *A. pyrophilus* SOD; *Hs*SOD, *H. salinarum* SOD; *Sa*SOD, *S. acidocaldarius* SOD; *Tt*SOD, *T. thermophilus* SOD; *Ec*SOD, *E. coli* Fe-SOD. The residues with high homology were highlighted by black background. (B) Crystal structure of *Ap*SOD (PDB ID 1COJ). The four subunits were drawn in different colors, and the *C-*terminal helix was presented in red. The last two turns of the *C-*terminal helix (residues from 202 to 210) were highlighted in blue, while K211 was in yellow.

Sequence alignment analysis indicated that the hyperthermostable TcSOD and *Ap*SOD are ~10 residues longer than the other less thermostable or mesophilic SODs (Figure 1A). These additional residues extend the *C-*terminal helix by two more helical turns and a coiled tail [17,18], which help to increase the interactions between the *C-*terminal helix and the neighboring subunit (Figure 1B). The stabilization interactions include four extra inter-subunit ion pairs (E194, K198, D208 and K211), extra hydrophobic interactions (A202, F209, V210 and L206) and an intra-subunit ion pair network (E204, K207, D208 and K211). Particularly, K211, the last residue at the *C-*terminus, forms a strong inter-subunit salt bridge with E194 (2.8 Å) at the A/C interface and a weak intra-subunit salt bridge with D208 (6.4 Å). In this research, the role of the extended *C-*terminus in the structural stability of TcSOD was investigated by study of the properties of two deletion mutants, M202 (residues 1–201) and M211 (residues 1–210). Unexpectedly, the removal of the last 10 residues at the *C-*terminus slightly decreased the stability of the tetrameric enzyme against inactivation and dissociation, but greatly destabilized the monomeric intermediate during TcSOD unfolding.

## 2. Results and Discussion

### 2.1. Characterization of the WT and Mutated TcSODs

**Figure 2.**
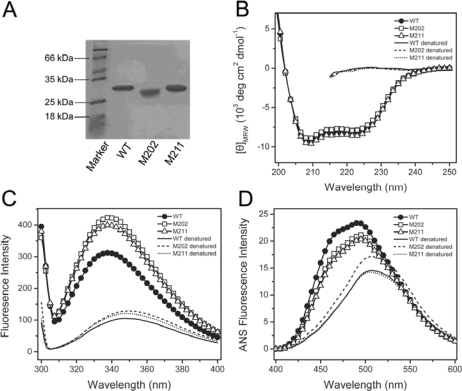
Characterization of the M211 and M202 mutants by SDS-PAGE (A), far-UV CD (B), intrinsic Trp fluorescence (C) and ANS fluorescence (D) spectra. The proteins were dissolved in 20 mM phosphate buffer, pH 7.4, with a final concentration of 0.27 mg/mL. The denatured samples were prepared by dissolving the enzymes in 20 mM phosphate buffer in the presence of 6 M GdnHCl for 12 h at 4 °C. The CD data were presented as the mean residue ellipticity ([*θ*]_MRW_). The intrinsic fluorescence was exited at 295 nm, and the ANS fluorescence was exited at 380 nm. All spectroscopic experiments were carried out at 25 °C.

The WT and mutated TcSODs were purified from the soluble fractions of the cell lysates, and the purity was evaluated by 12.5% SDS-PAGE (Figure 2A) and SEC experiments. A comparison of the biophysical and biochemical properties of the WT and mutants is summarized in Table 1. The SEC profiles for the three proteins were similar, which contains a single peak eluted at around 14.4 mL, a value similar to our previous result [10], implying that the mutation did not affect the oligomeric state of TcSOD. Enzymatic assay indicated that neither M211 nor M202 affected the catalytic activity of TcSOD, which is consistent with the fact that the *C-*terminus is far away from the active site. The effect of the mutations on the metal content of TcSOD was measured by inductively coupled plasma high resolution mass spectrometry. The metal content of the three proteins (0.37–0.47 per subunit) was similar to those reported previously for the WT (0.35–0.52 per subunit) [10,17]. It is worth noting that the iron content was smaller than 1 per subunit. This might be caused by metal loss during purification and dialysis for the mass spectrometry experiments [17], and has also been reported by other groups [14,18]. Actually, the slight difference in ion content (0.35–0.52 per subunit) did not significantly affect the spectroscopic and biochemical properties of TcSOD when evaluated by different lots of proteins (data not shown).

The effects of the mutations on TcSOD secondary and tertiary structures were evaluated by CD, intrinsic Trp fluorescence and extrinsic ANS fluorescence spectra of the WT and mutated proteins (Figure 2B and 2D). The CD spectra of the mutants were almost identical to that of the WT protein in both the shape and mean residue ellipticities at 208 and 222 nm, indicating that the mutants were well-structured. Meanwhile, the intrinsic Trp florescence spectra of the three proteins had the same maximum emission wavelength (*E*_m_) at about 337 nm, suggesting that the solvent-accessibility of the Trp residues was not affected by the mutations. However, both the M211 and M202 mutations resulted in a 30% increase of the Trp fluorescence intensity. This intensity increase might be due to the change of the fluorescence partially-quenched in the WT protein or an alternation of the flexibility of the residues around the Trp residues. Nonetheless, the intrinsic fluorescence results implied that the mutations might lead to a disturbance at the microenvironment around the Trp residues. A minor decrease in ANS fluorescence intensity was also observed for the two mutants, indicating that the hydrophobic exposure of TcSOD was slightly decreased by the mutations. These spectroscopic results suggested that the mutation had little effect on the secondary structure, but slightly modified the tertiary structure of TcSOD.

### 2.2. Thermal Stability of the WT and Mutated TcSODs

**Figure 3.**
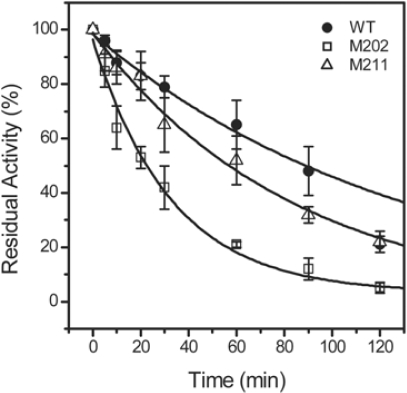
Thermal inactivation of the WT TcSOD, M202 and M211. The enzymes were dissolved in 20 mM phosphate buffer, pH 7.4, and heated at 95 °C. Aliquots of the enzyme solutions were taken at given time intervals, and then the residual activity was measured by the pyrogallol method at 25 °C. The final protein concentration was 0.27 mg/mL. The residual activity was normalized by taken the activity of the enzyme without heat-treatment as 100%. The data were fitted by the first-order kinetics, and the fitted data are presented as solid lines.

The effect of the mutations on the thermal stability of TcSOD was evaluated by measuring the time-course thermal inactivation at 80 °C or 95 °C. When incubated at 80 °C, all of the three proteins were stable, and maintained ~95% residual activity after 2 h incubation (data not shown). At 95 °C, the time when TcSOD lost half of its activity was 91, 58 and 23 min for the WT, M211 and M202, respectively (Figure 3).

The significant decrease in the thermal stability at 95 °C induced by the M202 mutation suggested that the *C-*terminal helix contributed to the hyperthemostability of TcSOD. The changes in the transition free energy of thermal inactivation (ΔΔ*G*^‡^_in_) by the mutations could be calculated from Equation (15), and it was −4.2 kJ/mol for M202 and −1.4 kJ/mol for M211.

### 2.3. GdnHCl-induced Inactivation and Equilibrium Unfolding GdnHCl-induced Inactivation and Equilibrium Unfolding

**Figure 4.**
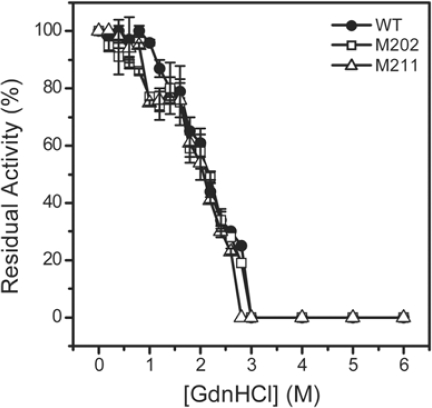
Inactivation of the WT and mutated TcSOD by GdnHCl. The enzymes were denatured in 20 mM phosphate buffer, pH 7.4, in the presence of various concentrations of GdnHCl for 12 h at 4 °C. The final protein concentration was 0.27 mg/mL. The residual activity was normalized by taken the activity of the enzyme in buffer without GdnHCl as 100%.

**Figure 5.**
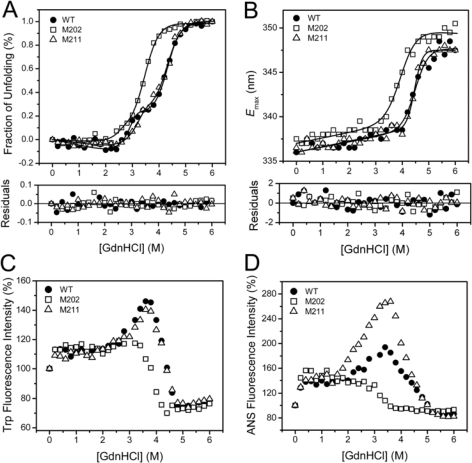
Unfolding of WT and mutants monitored by the ellipticity at 222 nm (A), emission maximum wavelength (B) and intensity (C) of the intrinsic fluorescence, and ANS fluorescence intensity at 470 nm (D). The data in panel A and B were fitted to a three-state transition and a two-state transition, respectively, using the equations listed in Materials and Methods. The CD data were normalized by (*F*–*F*_1_)/(*F*_0_–*F*_1_), where *F* is the ellipticity at 222 nm, *F*_0_ and *F*_1_ are the ellipticity of the native and fully-denatured proteins, respectively.

GdnHCl-induced inactivation and unfolding were performed to quantitatively evaluate the contributions of the extended *C-*terminal helix to TcSOD stability. For all transition curves, no significant difference was observed between the 0.27 and 0.7 mg/mL samples. This implied that the GdnHCl-induced denaturation was independent of protein concentration for all of the three proteins. The results of the 0.27 mg/mL sample were presented in Figures 4 and 5. All the three enzymes were fully-inactivated at GdnHCl concentrations above 3.0 M, and the midpoint of inactivation was at around 2.15 M GdnHCl (Figure 4). It is worth noting that the inactivation curves of the mutants slightly deviated form that of the WT enzyme and had a small platform between 1.0 M and 1.5 M GdnHCl. This might result from the stabilization of a tetrameric native-like intermediate, which was not obvious during the folding of the WT enzyme [10].

Consistent with the previous results [10], the GdnHCl-induced equilibrium unfolding of the WT TcSOD was dominated by a three-state process (N_4_ ↔ 4I ↔ 4U) when monitored by spectroscopic methods. It is worth noting that the involvement of a possible tetrameric intermediate was characterized by the inactivation experiments (Figure 4). However, this intermediate was unable to be detected by CD and fluorescence spectroscopy, and thus was not included in the following curve fitting analysis. The population of a molten globule-like intermediate (I) was characterized by the appearance of a small plateau in the transition curve from the CD data and a peak in the intrinsic and ANS fluorescence at around 3 M GdnHCl (Figure 5). The unfolding monitored by *E*_m_ was an apparent two-state process, which corresponded to the I→U transition as characterized previously [10].

The transition curves of M211 were almost identical to those of the WT protein, except that the maximum ANS fluorescence was much higher than that of the WT. This suggested that the deletion of the last residue at the *C-*terminus increased the hydrophobic exposure of the intermediate state. As for M202, great discrepancy from the WT was observed for all the transition curves. The CD and *E*_m_ data clearly indicated that the deletion of the last 10 residues at the *C-*terminus significantly decreased TcSOD stability against GdnHCl denaturation. Moreover, the transition curves from the intrinsic and ANS fluorescence intensity did not have a peak corresponding to the molten globule-like intermediate, which is quite different from the observations for the WT and M211. This phenomenon implied that the molten globule-like intermediate was unstable, and was not populated during the unfolding of M202.

A quantitative evaluation of the effect of the mutations on TcSOD unfolding was achieved by global fitting of the CD data using Equation 9. The thermodynamic parameters (Table 2) of the WT was similar to those reported elsewhere obtained by independent linear fitting [10]. The M211 mutation slightly decreased the overall stability of TcSOD (~20 kJ/mol for $\Delta G_{\text{NU}}^{{\text{H}}_{2}\text{O}}$). Both the $\Delta G_{\text{NI}}^{{\text{H}}_{2}\text{O}}$ and $\Delta G_{\text{IU}}^{{\text{H}}_{2}\text{O}}$ values of M211 were about 5 kJ/mol smaller than that of the WT, and these values were at the same level as the contribution of a hydrogen bond or an ion pair to protein stability characterized previously [19–21]. This result also indicated that the deletion of the last residue at the *C-*terminus affected both the N_4_→4I and I→U transitions. As for M202, the total Gibbs free energy $\Delta G_{\text{NU}}^{{\text{H}}_{2}\text{O}}$ was 70 kJ/mol smaller than that of the WT. A significant decrease was observed for both the $\Delta G_{\text{NI}}^{{\text{H}}_{2}\text{O}}$ and $\Delta G_{\text{IU}}^{{\text{H}}_{2}\text{O}}$ values, which were ~15 and ~14 kJ/mol smaller than those of the WT, respectively. These results suggested that the extended *C-*terminal helix in TcSOD contributed to the stability of both the native and intermediate state. It is worth noting that $\Delta G_{\text{NI}}^{{\text{H}}_{2}\text{O}}$ was much larger than $\Delta G_{\text{IU}}^{{\text{H}}_{2}\text{O}}$ for all of the three proteins. Thus the 5–15 kJ/mol decrease by the mutations did not significantly affect the N_4_→4I transition, but had a notable effect on the I→U transition.

### 2.4. Discussion

Thermophilic and thermostable enzymes are of particularly interest in exploring the molecular mechanisms of protein thermostability. Although the experimental data have been increasingly accumulated, no general rules are available yet for the explanation and prediction of the remarkable stability of the thermophilic proteins. Among the possible factors, ion-pairing has been extensively investigated by structural modeling and site-directed mutagenesis, and most studies support the idea that ion-pairing is a strong stabilizing factor for hyperthermophilc proteins [3,22–24]. For example, the introduction of new ion pairs has successfully been used as a tool to improve the thermostability of enzymes [3,24–27]. However, some reports indicated that ion-pairing can also be destabilizing or lead to thermolabile mutants [1,5,28]. Structural analysis indicates that the hyperthermophilic TcSOD and *Ap*SOD contain an increased number of inter- and intra-subunit ion pairs compared to the mesophilic SODs [17,18]. Particularly, the ~10 residues extended *C-*terminal tail forms an additional ion-pairing network through both inter- and intra-subunit ion pairs (Figure 1). In this research, mutational analysis indicated that the ion-pairing network at the *C-*terminus indeed contributed to the stabilization of TcSOD against heat- and GdnHCl-induced denaturation (Figures 3 and 5). The deletion mutations were found to destabilize TcSOD at 95°C by decreasing the ΔΔ*G*^‡^_in_ value (about 4.2 kJ/mol for M202 and 1.4 kJ/mol for M211). However, the mutations had no significant effects on TcSOD stability at 80 °C. Moreover, the mutations had no significant effects on TcSOD stability against GdnHCl-induced inactivation. These observations suggested that the additional ion-pairing network at the *C-*terminus had limited contributions to the extraordinary stability of TcSOD.

A common feature for hyperthermostable SODs from different resources, such as archaea and thermophilic bacterium, is that they usually exist in higher oligomeric states than their homologous mesophilic enzymes [29]. Oligomerization has been proposed to make a critical contribution to the stability of proteins [30–34], and the stability of the quaternary structure is extremely important to the hyperthermostability of archaeon proteins [9]. Structural analysis indicated that the extended *C-*terminus of the hyperthermophilic SODs stabilizes the enzymes by participating in the A/C subunit interface via intra- and inter-subunit ion pairs and hydrophobic interactions [17,18]. However, the dissociation of the tetrameric enzymes during unfolding was only slightly affected, and the $\Delta G_{\text{NI}}^{{\text{H}}_{2}\text{O}}$ value was decreased less than 10% by the mutations (Table 2). The unfolding results herein indicated that the *C-*terminal ion-pairing network was not crucial to the dissociation of TcSOD, suggesting that the other parts of the A/C interface might be much more important to TcSOD quaternary structural stability. This might also be the reason why the mutations were thermostable (Figure 3) and had minor effects on TcSOD inactivation induced by GdnHCl (Figure 4). Unlike its role in A/C dimer formation, little intra-subunit interactions could be characterized by structural analysis between the extended *C-*terminus and the central structure (Figure 1B). Unexpectedly, the most striking effect of the mutations on TcSOD unfolding was the great decrease in the $\Delta G_{\text{IU}}^{{\text{H}}_{2}\text{O}}$ value, which was 10% by M211 and 30% by M202 (Table 2). One possible explanation is that the two more helical turns extended by the last 10 residues at the *C-*terminus might help to stabilize the *C-*terminal helix, which is crucial to the I→U transition of TcSOD unfolding. Thus, the results herein suggested that the additional ion-pairing network mainly enhanced the structural stability of TcSOD by stabilizing the monomers. The observations that the *C-*terminal helix did not significantly affect the hyperthermostability might be correlated to its limited contributions to subunit interactions of TcSOD, which also highlight the roles of oligomerization in the extraordinary stability of extremophilic proteins.

## 3. Experimental Section

### 3.1. Materials

Guanidine hydrochloride (GdnHCl), 8-anilino-1-naphthalenesulfonic-acid (ANS) and sodium dodecyl sulfate (SDS) were purchased from Sigma Chemical Corporation. Restriction enzymes, *Taq* DNA polymerase and T4 DNA ligase were purchased from TaKaRa Biotechnology Co., Ltd. (Dalian). Vector pET28a was purchased from Novagen (Merck KGaA, Darmstadt, Germany). All other chemicals were local products of analytical grade.

### 3.2. Site-directed Mutagenesis

Two mutants of TcSOD, M202 and M211, in which A202 and K211 in TcSOD were replaced by the stop codon (TAA), were obtained by site-directed mutagenesis. The mutagenic primers were: 5′-CC*AAGCTT*TTACTTCATAGCCTTTTC-3′ for M202, and 5′-CC*AAGCTT*TTATTACACAAAATCCTT-3′ for M211 (the *BamH*I and *Hind*III sites are in italics). These two primers were used as the 3′-reverse primers in the polymerase chain reaction, and the forward primers were the same as those described previously [17] for TcSOD. Site-directed mutations were carried out using 10 ng of the double-strand DNA (entire plasmid vector harboring the *sod* gene), 10 pmol of the primer, *LA-Taq* DNA polymerase and the buffer supplied with the DNA polymerase. The 25 cycles of amplification was performed as follows: 94 °C for 30 s, 55 °C for 30 s, and 72 °C for 30 s. The amplified fragments were inserted into the vector pET28a after both digested with *BamH*I and *Hind*III. The ligated plasmids were verified by DNA sequencing.

### 3.3. Protein Expression, Purification and Characterization

The WT TcSOD and the two mutants were expressed in *E. coli* BL21 with pET28a plasmid and purified by Ni-NTA affinity chromatography and size-exclusion chromatography (SEC) as described previously [17]. The purity of final products was evaluated by 12.5% SDS- and 10% native-PAGE analysis. The protein concentrations and SOD activity was assayed according to the standard methods [35,36]. The SOD activity was defined as the amount of enzyme that inhibits the autoxidation of pyrogallol by 50% as described previously [35]. The metal contents of SODs were detected by inductively coupled plasma high resolution mass spectrometry at the Analytical Center of Tsinghua University using the samples after dialysis against the metal-ion-free buffer.

### 3.4. Size-exclusion Chromatography

The size-exclusion chromatography (SEC) experiments were carried out on a Superdex 200HR 10/30 column on an AKTA FPLC (Amersham Phamacia Biotech, Sweden). The column was pre-equilibrated with 20 mM sodium phosphate buffer (pH 7.4), and then 100 μL protein solutions were injected into the column. All samples were run at a flow rate of 0.5 mL/min at 25 °C.

### 3.5. Spectroscopic Experiments

All spectroscopic experiments were performed at 25 °C with a protein concentration of 0.27 mg/mL. Details regarding the spectroscopic measurements were the same as those described elsewhere [10]. In brief, the Far-UV circular dichroism (CD) spectra were recorded on a Jasco 715 spectrophotometer (Jasco Corp., Tokyo, Japan) and the fluorescence spectra were measured on an F-2500 spectrophotometer (Hitachi Ltd., Tokyo, Japan). The ANS binding affinity to the WT and the mutants was monitored with an excitation wavelength of 380 nm and an emission wavelength ranging from 400 to 600 nm.

### 3.6. Data Analysis

The equilibrium folding transition curve monitored by CD was analyzed according to a three-state model as characterized previously [10]:

(1) $${\text{N}}_{4}↔4\text{I}↔4\text{U}$$

where N_4_ is the native tetrameric protein, I is the monomeric intermediate state, and U is the fully-unfolded state. The equilibrium constants for the two transitions in Equation (1) are:

(2) $$K_{\text{NI}}={\left[\text{I}\right]}^{4}/[{\text{N}}_{4}],K_{\text{IU}}=[\text{U}]/[\text{I}]$$

and the mole fractions of each species are:

(3) $$f_{1}=[\text{I}]/P,f_{\text{N}}=4[{\text{N}}_{4}]/P=4P^{3}f_{\text{I}}^{4}/K_{\text{NI}},f_{\text{U}}=[\text{U}]/P=f_{\text{I}}K_{\text{IU}}$$

(4) $$f_{\text{N}}+f_{\text{I}}+f_{\text{U}}=4P^{3}f_{\text{I}}^{4}/K_{\text{NI}}+f_{\text{I}}+f_{\text{I}}K_{\text{IU}}=1$$

Then *f*_I_ can be obtained from Equation (4):

(5) $$f_{\text{I}}=-\frac{1}{2}\sqrt{-m+n}+\frac{1}{2}\sqrt{m-n+2n/m\sqrt{-m+n}}$$

in which:

$$\begin{array}{c} m=4{\left(\frac{1}{3}\right)}^{⅓}/({\left(9ab^{2}+\sqrt{3}\sqrt{256a^{3}+27a^{2}b^{4}}\right)}^{⅓})\\ n=({\left(9ab^{2}+\sqrt{3}\sqrt{256a^{3}+27a^{2}b^{4}}\right)}^{⅓})/2^{⅓}3^{⅔}a,a=4P^{3}/K_{\text{NI}},b=1+K_{\text{IU}} \end{array}$$

The free energy changes can be expressed as a function of the GdnHCl concentration:

(6) $$\Delta G_{\text{IU}}=-RT\text{ln}K_{\text{IU}}=\Delta G_{\text{IU}}^{H_{2}0}-m_{\text{IU}}[\text{GdnHcl}]$$

(7) $$\Delta G_{\text{NI}}=-RT\text{ln}K_{\text{NI}}=\Delta G_{\text{NI}}^{{\text{H}}_{2}0}-m_{\text{NI}}[\text{GdnHcl}]$$

(8) $$\Delta G_{\text{NU}}^{{\text{H}}_{2}0}=\Delta {\text{G}}_{\text{NI}}^{{\text{H}}_{2}0}+4\Delta {\text{G}}_{\text{IU}}^{{\text{H}}_{2}0}$$

The CD data are described as:

(9) $$y=f_{\text{N}}(y_{\text{N}}+m_{\text{N}}[\text{GdnHCl}])+f_{\text{I}}y_{\text{I}}+f_{\text{U}}(y_{\text{U}}+m_{\text{U}}[\text{GDnHCl}])$$

where *y* is the global relative ellipticity at 222 nm, *y*_N_ and *y*_U_ are the intercept of the initial and final baselines, respectively. *m*_N_ and *m*_U_ are the slopes of the initial and final baselines, respectively, and *y*_I_ represents the fraction of the intermediate calculated by the CD data. The folding profiles were fitted to Equation (9) with the regression wizard of SigmaPlot, followed by the nonlinear least-squares algorithm.

The equilibrium folding transition curve monitored by *E*_max_ was analyzed according to a two-state model:

(10) $${\text{N}}_{4}↔4\text{U}$$

The mole fractions of N and U are:

(11) $$K_{\text{NU}}={\left[\text{U}\right]}^{4}/[{\text{N}}_{4}]$$

(12) $$f_{\text{N}}=4[{\text{N}}_{4}]/P=4P^{3}f_{\text{U}}^{4}/K_{\text{NU}},f_{\text{U}}=[\text{U}]/P=f_{\text{U}}K_{\text{NU}}$$

(13) $$f_{\text{N}}+f_{\text{U}}=4P^{3}f_{\text{U}}^{4}/K_{\text{NU}}+f_{\text{U}}K_{\text{KU}}=1$$

The root of Equation (13) is the same as that described in Equation (5), except for: *a* = 4*P*^3^/*K*_NU_ and *b* = 1. Thus the free energy changes can be expressed as:

(14) $$\Delta G_{\text{NU}}=-RT\text{ln}K_{\text{NU}}=\Delta G_{\text{NU}}^{{\text{H}}_{2}0}=m_{\text{NU}}[\text{GdnHCl}]$$

### 3.7. Thermal- and GdnHCl- Induced Inactivation

The thermostability of SODs were measured by treating the enzymes at 95 °C in 20 mM sodium phosphate buffer, pH 7.4, and aliquots of the enzyme solutions were taken at given time intervals. Then the residual activity was measured by the pyrogallol method at 25 °C [35]. The inactivation rate constants (*k*_in_) were obtained by fitting the thermal inactivation data by the first-order kinetics. The changes in the transition free energy of thermal inactivation (ΔΔ*G*^‡^_in_) was calculated according to the Equation (15) [37]:

(15) $$\Delta \Delta G_{\text{in}}^{‡}=\text{RTln}(k_{\text{in},\text{WT}}/k_{\text{in},\text{mutant}})$$

GdnHCl inactivation was carried out by dissolving the enzymes in 20 mM sodium phosphate buffer, pH 7.4, containing various concentrations of GdnHCl for 12 h at 4 °C. The final concentration of the protein was 0.27 mg/mL for both the thermal- and GdnHCl-inactivation experiments. The residual activity was normalized by taking the activity of the sample treated at 25 °C in the absence of GdnHCl as 100%.

## 4. Conclusions

In this research, we investigated the impact of the extended *C-*terminal helix on the activity, structure, and stability of TcSOD. A comparison of the properties of the WT enzyme and two deletion mutants indicated that the extra ion-pairing network at the *C-*terminus had limited contributions to the stability of TcSOD against heat- and GdnHCl-induced inactivation. Interestingly, the intactness of the *C-*terminal helix had dissimilar effects on the two stages of TcSOD unfolding. The mutations resulted in a minor decrease in the Gibbs free energy change of the TcSOD dissociation, while a significant decrease in that of the unfolding of the monomeirc intermediate. These results herein suggested that the additional ion-pairing network at the *C-*terminus mainly enhanced the structural stability of hyperthermophilic SODs by stabilizing the molten globule-like folding intermediate.

## Figures and Tables

**Table 1.** Characterization of the purified WT TcSOD and the mutants.

	**Substituted residue**	**Fe content per subunit**	**Specific activity (U/mg)**	**Elution volume (mL)**
WT	-	0.47	1,383 ± 108	14.41
M202	A202→stop	0.37	1,320 ± 37	14.45
M211	K211→stop	0.47	1,434 ± 98	14.28

**Table 2.** Thermodynamic parameters of the three-state unfolding of the WT TcSOD and the mutants.

	$\Delta G_{\text{NI}}^{{\text{H}}_{2}\text{O}}$ (kJ/mol)	*m*_NI_ (kJ/mol M)	$\Delta G_{\text{IU}}^{{\text{H}}_{2}\text{O}}$ (kJ/mol)	*m*_IU_ (kJ/mol M)	$\Delta G_{\text{NU}}^{{\text{H}}_{2}\text{O}}$ (kJ/mol)
WT	170 ± 18	30 ± 6	46 ± 3	10.8 ± 0.7	355 ± 28
M202	156 ± 5	30 ± 2	32 ± 2	9.3 ± 0.5	285 ± 12
M211	166 ± 17	26 ± 5	42 ± 3	9.5 ± 0.7	332 ± 29

## References

[1] Vieille C Zeikus GJ Hyperthermophilic enzymes: Sources, uses, and molecular mechanisms for thermostability Microbiol. Mol. Biol. Rev 2001 65 1 43 11238984 10.1128/MMBR.65.1.1-43.2001 PMC99017

[2] Scandurra R Consalvi V Chiaraluce R Politi L Engel PC Protein thermostability in extremophiles Biochimie 1998 80 933 941 9893953 10.1016/s0300-9084(00)88890-2

[3] Vetriani C Maeder DL Tolliday N Yip KS Stillman TJ Britton KL Rice DW Klump HH Robb FT Protein thermostability above 100 °C: A key role for ionic interactions Proc Natl Acad Sci USA 1998 95 12300 12305 9770481 10.1073/pnas.95.21.12300 PMC22826

[4] Unsworth LD van der Oost J Koutsopoulos S Hyperthermophilic enzymes-stability, activity and implementation strategies for high temperature applications FEBS J 2007 274 4044 4056 17683334 10.1111/j.1742-4658.2007.05954.x

[5] Kumar S Nussinov R How do thermophilic proteins deal with heat? Cell. Mol. Life Sci 2001 58 1216 1233 11577980 10.1007/PL00000935 PMC11337401

[6] Grabarse W Vaupel M Vorholt JA Shima S Thauer RK Wittershagen A Bourenkov G Bartunik HD Ermler U The crystal structure of methenyltetrahydromethanopterin cyclohydrolase from the hyperthermophilic archaeon *Methanopyrus kandleri* Structure 1999 7 1257 1268 10545331 10.1016/s0969-2126(00)80059-3

[7] Ursby T Adinolfi BS Al-Karadaghi S de Vendittis E Bocchini V Iron superoxide dismutase from the archaeon *Sulfolobus solfataricus*: Analysis of structure and thermostability J. Mol. Biol 1999 286 189 205 9931259 10.1006/jmbi.1998.2471

[8] Thoma R Hennig M Sterner R Kirschner K Structure and function of mutationally generated monomers of dimeric phosphoribosylanthranilate isomerase from *Thermotoga maritima* Structure 2000 8 265 276 10745009 10.1016/s0969-2126(00)00106-4

[9] Tanaka Y Tsumoto K Yasutake Y Umetsu M Yao M Fukada H Tanaka I Kumagai I How oligomerization contributes to the thermostability of an archaeon protein. Protein l-isoaspartyl-*O*-methyltransferase from *Sulfolobus tokodaii* J. Biol. Chem 2004 279 32957 32967 15169774 10.1074/jbc.M404405200

[10] Wang S Liu W-F He Y-Z Zhang A Huang L Dong Z-Y Yan Y-B Multistate folding of a hyperthermostable Fe-superoxide dismutase (TcSOD) in guanidinium hydrochloride: The importance of the quaternary structure Biochim. Biophys. Acta: Prot. Proteom 2008 1784 445 454 10.1016/j.bbapap.2007.12.001 18166165

[11] Brookes PS Yoon YS Robotham JL Anders MW Sheu SS Calcium, ATP, and ROS: A mitochondrial love-hate triangle Am. J. Physiol.: Cell Physiol 2004 287 C817 C833 15355853 10.1152/ajpcell.00139.2004

[12] Fridovich I Superoxide radical and superoxide dismutases Annu. Rev. Biochem 1995 64 97 112 7574505 10.1146/annurev.bi.64.070195.000525

[13] Miller A-F Superoxide dismutases: Active sites that save, but a protein that kills Curr. Opin. Chem. Biol 2004 8 162 168 15062777 10.1016/j.cbpa.2004.02.011

[14] Lim J-H Yu YG Choi I-G Ryu J-R Ahn B-Y Kim S-H Han Y-S Cloning and expression of superoxide dismutase from *Aquifex pyrophilus*, a hyperthermophilic bacterium FEBS Lett 1997 406 142 146 9109405 10.1016/s0014-5793(97)00262-7

[15] Knapp S Kardinahl S Hellgren N Tibbelin G Schäfer G Ladenstein R Refined crystal structure of a superoxide dismutase from the hyperthermophilic archaeon *Sulfolobus acidocaldarius* at 2.2 Å resolution J. Mol. Biol 1999 285 689 702 9878438 10.1006/jmbi.1998.2344

[16] Whittaker MM Whittaker JW Recombinant superoxide dismutase from a hyperthermophilic archaeon, *Pyrobaculum aerophilium* J. Biol. Inorg. Chem 2000 5 402 408 10907751

[17] He Y-Z Fan K-Q Jia C-J Wang Z-J Pan W-B Huang L Yang K-Q Dong Z-Y Characterization of a hyperthermostable Fe-superoxide dismutase from hot spring Appl. Microbiol. Biotechnol 2007 75 367 376 17262208 10.1007/s00253-006-0834-3

[18] Lim J-H Yu YG Han YS Cho S-J Ahn B-Y Kim S-H Cho Y The crystal structure of a Fe-superoxide dismutase from the hyperthermophile *Aquifex pyrophilus* at 1.9 Å resolution: Structural basis for thermostability J. Mol. Biol 1997 270 259 274 9236127 10.1006/jmbi.1997.1105

[19] Horovitz A Serrano L Avron B Bycroft M Fersht AR Strength and co-operativity of contributions of surface salt bridges to protein stability J. Mol. Biol 1990 216 1031 1044 2266554 10.1016/S0022-2836(99)80018-7

[20] Bycroft M Fersht AR Surface electrostatic interactions contribute little to stability of barnase J. Mol. Biol 1991 220 779 788 1870131 10.1016/0022-2836(91)90117-o

[21] Shirley BA Stanssens P Hahn U Pace CN Contribution of hydrogen bonding to the conformational stability of ribonuclease T1 Biochemistry 1992 31 725 732 1731929 10.1021/bi00118a013

[22] Yip KS Stillman TJ Britton KL Artymiuk PJ Baker PJ Sedelnikova SE Engel PC Pasquo A Chiaraluce R Consalvi V Scandurra R Rice DW The structure of *Pyrococcus furiosus* glutamate dehydrogenase reveals a key role for ion-pair networks in maintaining enzyme stability at extreme temperatures Structure 1995 3 1147 1158 8591026 10.1016/s0969-2126(01)00251-9

[23] Vogt G Woell S Argos P Protein thermal stability, hydrogen bonds, and ion pairs J. Mol. Biol 1997 269 631 643 9217266 10.1006/jmbi.1997.1042

[24] Karshikoff A Ladenstein R Ion pairs and the thermotolerance of proteins from hyperthermophiles: A “traffic rule” for hot roads Trends Biochem. Sci 2001 26 550 556 11551792 10.1016/s0968-0004(01)01918-1

[25] Leemhuis H Rozeboom HJ Dijkstra BW Dijkhuizen L Improved thermostability of *Bacillus circulans* cyclodextrin glycosyltransferase by the introduction of a salt bridge Proteins 2004 54 128 134 14705029 10.1002/prot.10516

[26] Bae E Phillips GN Jr Identifying and engineering ion pairs in adenylate kinases. Insights from molecular dynamics simulations of thermophilic and mesophilic homologues J. Biol. Chem 2005 280 30943 30948 15995248 10.1074/jbc.M504216200

[27] Sadeghi M Naderi-Manesh H Zarrabi M Ranjbar B Effective factors in thermostability of thermophilic proteins Biophys. Chem 2006 119 256 270 16253416 10.1016/j.bpc.2005.09.018

[28] Lim JH Hwang KY Choi J Lee DY Ahn BY Cho Y Kim KS Han YS Mutational effects on thermostable superoxide dismutase from Aquifex pyrophilus: Understanding the molecular basis of protein thermostability Biochem. Biophys. Res. Commun 2001 288 263 268 11594783 10.1006/bbrc.2001.5752

[29] Schafer G Kardinahl S Iron superoxide dismutases: Structure and function of an archaic enzyme Biochem. Soc. Trans 2003 31 1330 1334 14641056 10.1042/bst0311330

[30] Villeret V Clantin B Tricot C Legrain C Roovers M Stalon V Glansdorff N van Beeumen J The crystal structure of *Pyrococcus furiosus* ornithine carbamoyltransferase reveals a key role for oligomerization in enzyme stability at extremely high temperatures Proc. Natl. Acad. Sci. USA 1998 95 2801 2806 9501170 10.1073/pnas.95.6.2801 PMC19649

[31] Shima S Thauer RK Ermler U Durchschlag H Tziatzios C Schubert D A mutation affecting the association equilibrium of formyltransferase from the hyperthermophilic *Methanopyrus kandleri* and its influence on the enzyme’s activity and thermostability Eur. J. Biochem 2000 267 6619 6623 11054114 10.1046/j.1432-1327.2000.01756.x

[32] Walden H Bell GS Russell RJ Siebers B Hensel R Taylor GL Tiny TIM: A small, tetrameric, hyperthermostable triosephosphate isomerase J. Mol. Biol 2001 306 745 757 11243785 10.1006/jmbi.2000.4433

[33] Ogasahara K Khechinashvili NN Nakamura M Yoshimoto T Yutani K Thermal stability of pyrrolidone carboxyl peptidases from the hyperthermophilic *Archaeon*, *Pyrococcus* furiosus Eur. J. Biochem 2001 268 3233 3242 11389725 10.1046/j.1432-1327.2001.02220.x

[34] Feng S Zhao T-J Zhou H-M Yan Y-B Effects of the single point genetic mutation D54G on muscle creatine kinase activity, structure and stability Int. J. Biochem. Cell Biol 2007 39 392 401 17030001 10.1016/j.biocel.2006.09.004

[35] Marklund S Marklund G Involvement of the superoxide anion radical in the autoxidation of pyrogallol and a convenient assay for superoxide dismutase Eur. J. Biochem 1974 47 469 474 4215654 10.1111/j.1432-1033.1974.tb03714.x

[36] Bradford MM Rapid and sensitive method for the quantitation of microgram quantities of protein utilizing the principle of protein-dye binding Anal. Biochem 1976 73 248 254 942051 10.1016/0003-2697(76)90527-3

[37] Tams JW Welinder KG Unfolding and refolding of *Coprinus cinereus* peroxidase at high pH, in urea, and at high temperature. Effect of organic and ionic additives on these processes Biochemistry 1996 35 7573 7579 8652538 10.1021/bi953067l

